# Industry 4.0 Technologies Applied to the Rail Transportation Industry: A Systematic Review

**DOI:** 10.3390/s22072491

**Published:** 2022-03-24

**Authors:** Camilo Laiton-Bonadiez, John W. Branch-Bedoya, Julian Zapata-Cortes, Edwin Paipa-Sanabria, Martin Arango-Serna

**Affiliations:** 1 Facultad de Minas, Universidad Nacional de Colombia Sede Medellín, Medellín 050041, Colombia; jwbranch@unal.edu.co (J.W.B.-B.); mdarango@unal.edu.co (M.A.-S.); 2 Fundación Universitaria CEIPA, Sabaneta 055450, Colombia; julian.zapata@ceipa.edu.co; 3 Cotecmar, Cartagena 130001, Colombia; epaipa@cotecmar.com

**Keywords:** industry 4.0, railway industry, systematic literature review, technology, artificial intelligence, internet of things

## Abstract

Background: Industry 4.0 technologies have been widely used in the railway industry, focusing mainly on maintenance and control tasks necessary in the railway infrastructure. Given the great potential that these technologies offer, the scientific community has come to use them in varied ways to solve a wide range of problems such as train failures, train station security, rail system control and communication in hard-to-reach areas, among others. For this reason, this paper aims to answer the following research questions: what are the main issues in the railway transport industry, what are the technologic strategies that are currently being used to solve these issues and what are the technologies from industry 4.0 that are used in the railway transport industry to solve the aforementioned issues? Methods: This study adopts a systematic literature review approach. We searched the Science Direct and Web of Science database inception from January 2017 to November 2021. Studies published in conferences or journals written in English or Spanish were included for initial process evaluation. The initial included papers were analyzed by authors and selected based on whether they helped answer the proposed research questions or not. Results: Of the recovered 515 articles, 109 were eligible, from which we could identify three main application domains in the railway industry: monitoring, decision and planification techniques, and communication and security. Regarding industry 4.0 technologies, we identified 9 different technologies applied in reviewed studies: Artificial Intelligence (AI), Internet of Things (IoT), Cloud Computing, Big Data, Cybersecurity, Modelling and Simulation, Smart Decision Support Systems (SDSS), Computer Vision and Virtual Reality (VR). This study is, to our knowledge, one of the first to show how industry 4.0 technologies are currently being used to tackle railway industry problems and current application trends in the scientific community, which is highly useful for the development of future studies and more advanced solutions. Funding: Colombian national organizations Minciencias and the Mining-Energy Planning Unit.

## 1. Introduction

The railway industry is one of the most important industries related to the economic growth and commutation transport in countries. As the world is experiencing major changes in industry processes due to new developments in our technology, the digitization of the railway industry must go hand in hand with these technological advances given its importance in the economic development of countries. The use of new technologies such as Artificial Intelligence (AI), Internet of Things (IoT) and Cloud Computing in industry processes have the power of transforming productivity, employment and other facets related to the human economy [1].

At the present time, industry 4.0 technologies are the ultimate result of technical progress in information and communication technologies (ICTs) [2]. These technologies are able to make a more efficient use of resources and information, improving industry processes [3] and unlocking new opportunities in the development of new products [2]. In fact, industry 4.0 is built on technologies that allow communication environment, environmental identification, extensive data collection and custom instrument decisions [4], which are crucial in the optimization and maintenance of railway industry processes and infrastructure.

These technologies have been applied in many railway industry solutions that are mostly oriented to monitor and control the rail and train infrastructure. However, due to the large amount of research focused on the development of new tools based on industry 4.0 technologies in this field, it is imperative to understand which are the main problems that the scientific community is facing in the railway industry and how these technologies are implemented to solve these issues.

Because of this, this paper aims to provide an overview of the research work reported in this field and related to industry 4.0 technologies by means of a Systematic Literature Review (SLR). This will give a better overview of the applied industry 4.0 technologies for specific problems to assist other researchers in the development of more advanced solutions for the railway industry. Therefore, the following research questions are considered for the present SLR:

- What are the main issues in the railway transport industry?
- What are the technologic strategies that are currently being used to solve these issues?
- What are the industry 4.0 technologies used in the railway transport industry that are used to solve the aforementioned issues?

This paper is organized as follows: Section 2 describes the applied methodology where authors state the followed protocol that allowed us to extract the relevant data. Section 3 shows the obtained results from the systematic literature review and aims to answer the research questions. Section 4 includes the main limitations of this study. Finally, Section 5 constitutes the main conclusions as well as future research.

## 2. Methodology

### 2.1. Elegibility Criteria

This step aimed to stablish an inclusion and exclusion criteria for the extracted studies from the electronic databases and to select the ones that were more relevant to answer the proposed research questions. Therefore, we proposed the following for the inclusion criteria:

- Studies published from January 2017 to November 2021.
- Studies written in English or Spanish.
- Studies published in conferences or journals reviewed by academic peers.

Regarding the exclusion criteria, we analyzed the extracted articles in two steps. The first one was focused on analyzing each article by its title, abstract and keywords where we removed the ones that did not answer any of the three proposed research questions. Meanwhile, the second step was oriented to the full lecture of the articles. In the second step we found articles that, after a detailed lecture, did not give any pertinent information about industry 4.0 technologies in rail transportation that could lead towards the answer to the proposed research questions. Additionally, we excluded duplicate articles found in the multiple searches.

### 2.2. Information Sources

For the search information phase, we used some of the most important digital libraries oriented to technology and engineering that are currently available. The selected information sources are described in Table 1.

These information sources allow the use of search algorithms composed of logical operators that are useful in extracting the desired information to perform the systematic review.

### 2.3. Search Strategy

This phase was focused on the definition of the keywords that could help us extract all the available information in the selected scientific databases with the goal of leading us towards the answers to the proposed research questions. In order to do this, we created three keywords’ groups that are described in Table 2.

The first keywords group was oriented to the railway transportation activity. The second group was used as a complementary keywords group that includes multiple general words related to technologies that are currently being used in railway transportation solutions. Lastly, the third group contains industry 4.0 technologies related keywords to complement the search.

Afterwards, we created multiple search queries combining different groups using logical and Boolean operators. The search queries were made to find studies having at least one of the keywords belonging to the selected groups for that specific query. Therefore, we combined group 1 and group 2 for the first search query and group 1 and group 3 for the second one. These search queries are described in Table 3, where we share the used search query per information source.

It is important to mention that some digital libraries have restrictions in the number of words used in the search algorithm. For example, Science Direct limits the number of words to 8.

The use of these search algorithms let us recover a total of 515 articles from the selected electronic databases where 393 were from Science Direct and 122 from Web of Science. The protocol utilized in this systematic review [7] offers certain advantages. First, it speeds up the search for domains that have limited available literature, allowing for rapid identification of recognized authors and research centers within that specific domain. It also allows one to quickly identify technologies or groups of technologies used for further, more detailed analysis.

### 2.4. Selection and Collection Process

The search process was conducted using the words from the groups in Table 2 to define the algorithm search queries given in Table 3 used in the digital libraries.

The articles search process was limited to title, abstract and keywords in the platform of Science Direct. In the platform of Web of Science, it was limited to title and abstract. This is because we found that the words “train” and “transport” used in the search inside the abstract for Web of Science could lead to multiple results that do not necessarily lead to our topics of interest but that are useful in the title and keywords search for this engine. This search process was carried out by one researcher.

**Figure 1:**
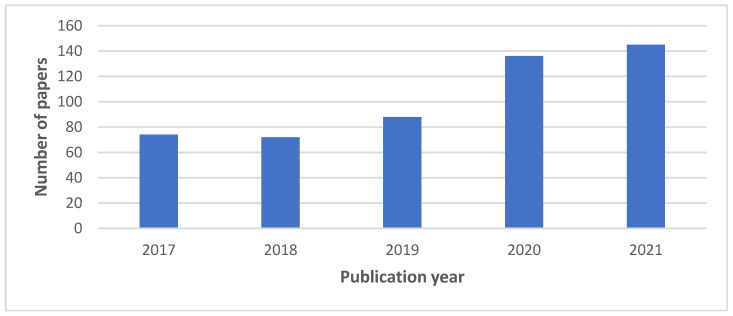
Distribution of the extracted papers by publication year.

Initially, we recovered 515 articles from electronic databases where 393 were from Science Direct and 122 from Web of Science. In Figure 1 we can see the number of published papers per year that were recovered from the initial search phase. Afterwards, we removed duplicated studies, and two researchers conducted the eligibility criteria based on the title, abstract and keywords to address the research questions where they discussed the articles that could be used for further processing. This process left us 201 studies, with 97 from Science Direct and 104 from Web of Science.

**Figure 2:**
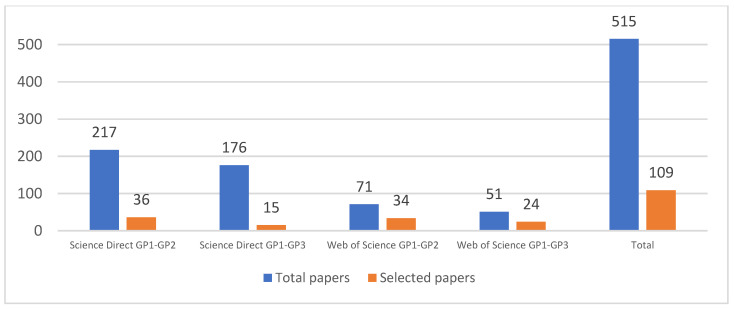
Comparison of the number of selected papers by search group.

Based on the eligibility criteria for Science Direct, we found multiple papers related to machine learning in the transport field because of the use of the word “train” (i.e., autonomous cars, highways object detection, etc.). After that, we reviewed each article that passed the initial eligibility criteria with a detail lecture to detect the most important technologies used in these solutions. This was done by pairs of researchers, and a consensus was reached by discussion. In the process, we detected articles that did not use any technology related to industry 4.0 and, therefore, were excluded. This process left us 109 studies with 52 studies from Science Direct and 57 from Web of Science. In Figure 2, we show the number of selected articles for the systematic review per database group.

## 3. Results

**Figure 3:**
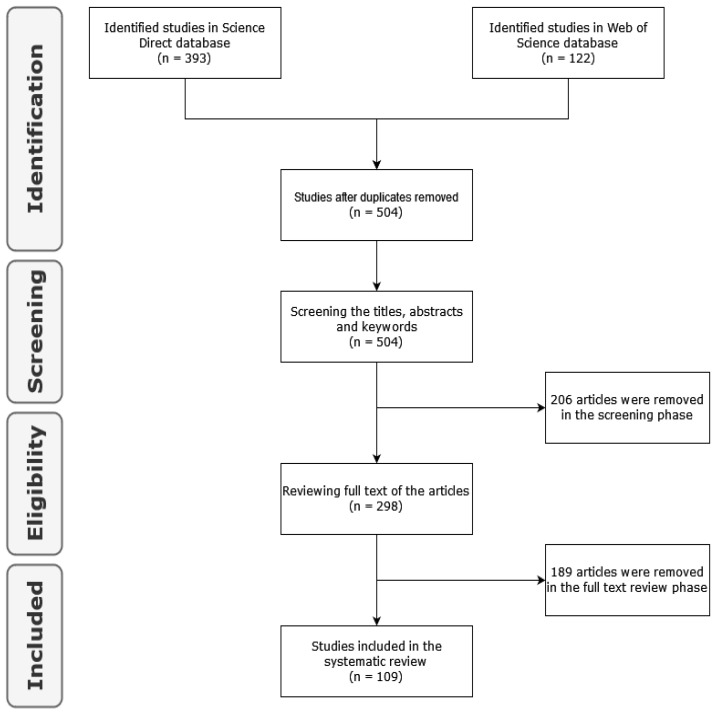
Summary review protocol.

This phase presents the results of the Systematic Literature Review (SLR) in order to answer the aforementioned research questions based on the main studies selected. The selected studies are the result of the search and selection process that was described in Section 2. In Figure 3 we can see a summary of the review protocol with the number of articles excluded in each phase and the final number of selected studies.

### 3.1. Answer to the First Research Question

**Figure 4:**
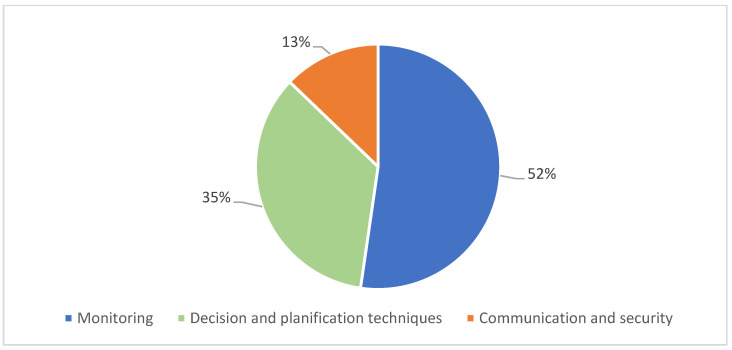
Distribution of the selected papers by application domain.

In order to determine the main issues in the railway transport industry and technological solutions, the extracted and filtered studies were classified into multiple groups that included the following: (i) monitoring, (ii) communication and security and (iii) decision and planification techniques. From these groups, the monitoring domain has the majority of the studies (52%) followed by decision and planification techniques (35%) and communication and security (13%) (see Figure 4). The classified articles can be seen in Table 4 where we show the application domain and subdomain they belong to.

#### 3.1.1. Monitoring Domain

Papers grouped in the monitoring domain are mostly focused on monitoring the railway infrastructure concerning technological assets, detecting irregularities in the rails and train equipment and environmental pollution monitoring, among others. It is important to mention that most of these papers aim to detect, as early as possible, any problem in the railway or train infrastructure in order to maintain the safety of train travel and reduce maintenance costs [117].

In fact, one of the main issues in train logistics for freight transport is related to the maintenance costs of the railway infrastructure. Usually, the maintenance costs are elevated due to contact measurement techniques and human inspections that are still used [118].

Hence, the use of real-time monitoring in the railway infrastructure is able to increase the reliability, availability, maintainability and security in the rail system [43].

**Figure 5:**
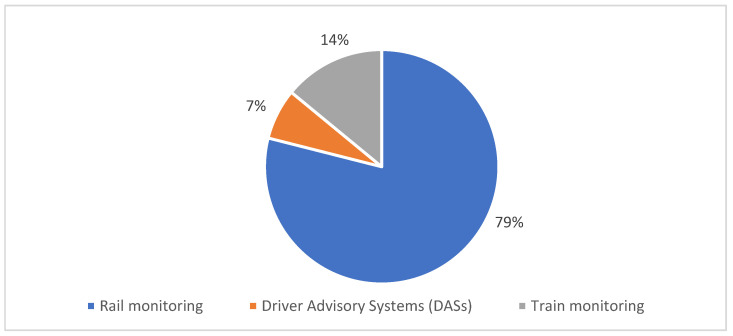
Distribution of the selected papers by monitoring domain.

The technological solutions included in the monitoring domain vary greatly in their end goal. For example, some of the technological solutions belonging to this group are focused on helping the train driver make better decisions based on collected train travel information, while others are more focused on monitoring the train equipment in order to prevent mechanical system faults. Therefore, the collected papers in this domain can be categorized into the following subdomains: rail monitoring (79%), Driver Advisory Systems (DASs) (7%) and train monitoring (14%) (see Figure 5).

It is necessary to highlight that most of the selected studies in this SLR can be categorized in more than one subdomain due to their multiple advantages of use in the train transport industry. Some of the most representative examples of railway system solutions based on industry 4.0 that are grouped into the monitoring domain are described below:

- *Rail monitoring*: Papers classified in this subdomain aimed to use technologies from industry 4.0 to check the state of the rail and its components to detect possible rail faults, decreasing maintenance costs and preventing railway accidents, among others. In fact, railroad system infrastructure is composed of a superstructure and a substructure [9]. The superstructure is usually composed of rails, sleepers and fastening systems, while the substructure is composed of subgrades, ballast and sub-ballasts. Monitoring systems can be present in two stages of the railroad lifetime. The first one is related to the manufacturing of railway equipment, and the second one is related to when the equipment is already installed in the railroad. Commonly, when monitoring systems are used in the fabrication of railway equipment it mostly focuses on achieving a zero-defect production. On the other hand, once the equipment is installed in the railroads, monitoring systems are responsible for constantly checking the behavior of the mechanical components and providing information to intelligent systems in order to prevent possible faults and risks in the train travel. Authors in [42] proposed a deep-learning-based model to automate railway wheelset inspection to improve the reliability and efficiency of traditional manual-based inspection protocols of wheelset assembly quality. The proposed neural network architecture, which is based on ResNet-50 [119] with a Siamese structure approach, uses images of 400 × 602 pixels as input and could achieve the 100% of the ground truth predictions on the test set of 3863 images, and has already been deployed in a manufacturing site.
- *Driver Advisory Systems (DAS’s)*: Studies classified in this domain proposed algorithms oriented to support train drivers along the travel of the train. For example, authors in [53] proposed an algorithm for a DAS’s that could help freight train drivers merge smoothly in merging areas where there could be multiple trains at the same time (freight and passenger trains) in order to avoid unnecessary stops and delays. The proposed algorithm was implemented as a mobile application named AftelAPP, which works in real-time and was tested in the Amsterdam Westhaven area, achieving good results.
- *Train monitoring*: Papers included in this subdomain are oriented to collecting train information in order to process it and gain insights about train performance, train pollution or train faults in the equipment. Usually, proposed studies in this subcategory collect and process train information in real time to improve daily operations and profit. Authors in [60] proposed a monitoring freight-train system using wireless sensors, IoT and a web-server application as a visualization tool. This solution called FEDORATA system can track freight-train parameters (geographical position, vibration, temperature, velocity, fuel or electricity consumption, etc.) to support administrative and technical decisions that could reduce maintenance costs.

#### 3.1.2. Communication and Security Domain

**Figure 6:**
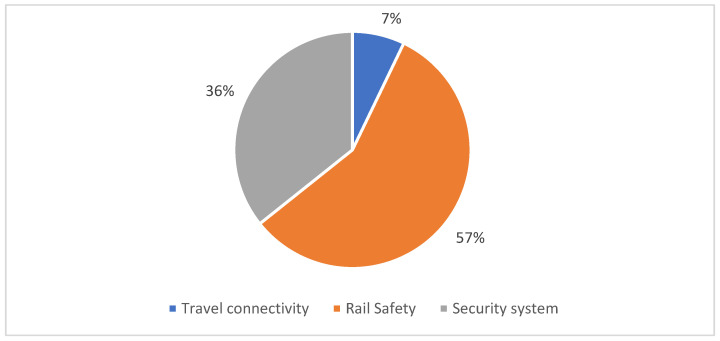
Distribution of the selected papers by communication and security domain.

Papers classified under communication and security domain were focused on the development of Industry 4.0 solutions for the improvement of security protocols of trains, train stations and railroads. Also, this subdomain focuses on papers that improve travel connectivity of trains. Generally, poor train travel connectivity is an important field of research due to its complexity that includes geographical location-based solutions, solutions for high-speed trains, underground railroads, among others. Technological solutions comprised in this domain can be classified in multiple subdomains such as rail safety (57%), security system (36%) and travel connectivity (7%) (see Figure 6).

**Figure 7:**
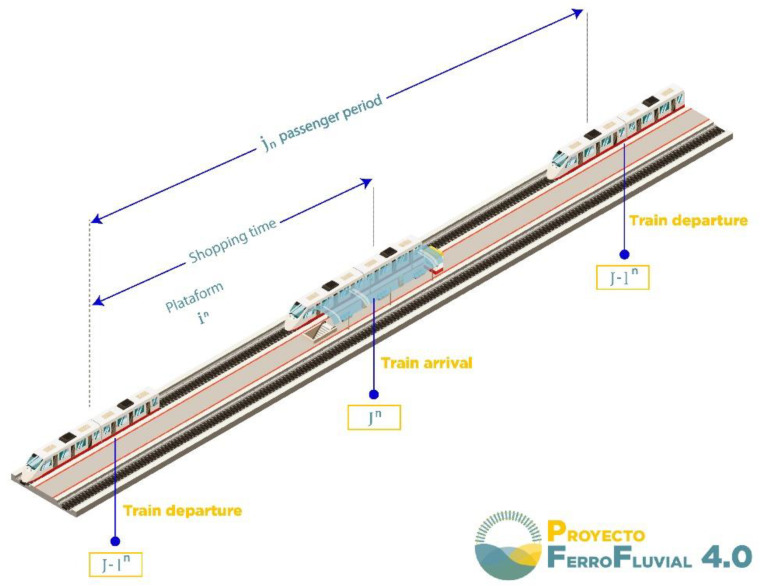
Diagram of an ideal train platform for passenger flow modified from [65].

**Figure 8:**
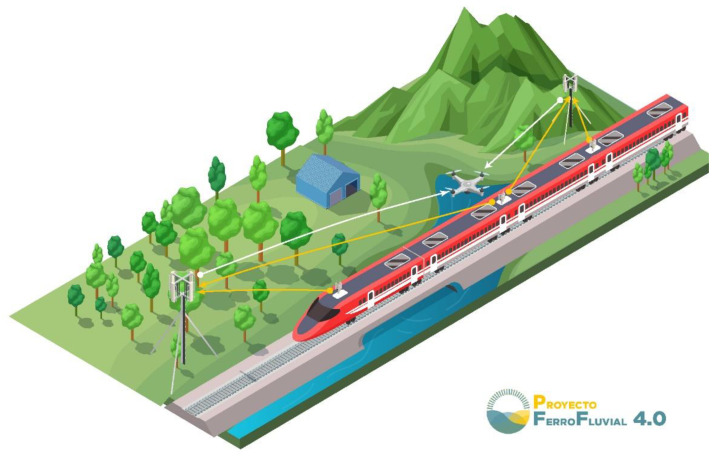
Proposed travel connectivity architecture in [78].

The aforementioned subdomains are characterized below:

- *Railway safety*: Papers included in this subdomain use industry 4.0 technologies to improve safety protocols in railroads and train stations using, mostly, a real-time risk status approach. For instance, an artificial neural network (ANN) in conjunction with a fuzzy system architecture was used to develop a smart risk-management system that could monitor overcrowding in train station areas [65] (see Figure 7). It is worth highlighting that in train stations the real-time monitoring of pedestrian behavior and movement flow is important to prepare safety measures to mitigate congestion in train station areas and avoid possible accidents such as stampedes [120].

- *Security systems*: Studies arranged in this subdomain focus on enhancing access protocols in railway communications, train station areas and railroads. Certainly, with the extensive deployment of technological assets such as IoT tools in railway systems or railway station network communications, the task of improving security protocols has become of vital importance. Accordingly, the use of AI cameras and Graphic Processing Units (GPUs) has been growing in railway security systems such as in [76]. The main goal of this study was to develop a real-time surveillance system for railway crossing using deep-learning models with GPU and images as input. In this approach, they designed an architecture that uses camera modules to capture real-time image information and send this data to a server to be processed. Additionally, their system architecture includes several security and privacy measures in order to secure all communication interfaces, protect personal data and increase personal privacy.
- *Travel connectivity*: Papers included in this subdomain aim to provide high data connectivity rates to passengers in trains while they are traveling. In fact, authors in [78] proposed a new concept named Travel Hopping Enabled Resource Allocation (THEResA), which is able to provide high data-rate connectivity in 5G+/6G to train passengers using unmanned aerial vehicles (UAVs) or drones. The proposed architecture with UAVs for this study is seen in Figure 8.

#### 3.1.3. Decision and Planification Techniques Domain

**Figure 9:**
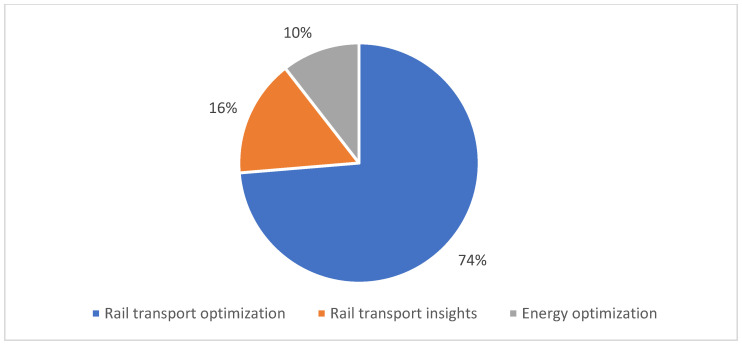
Distribution of the selected papers by decision and planification techniques domain.

The last domain used to classify the previously filtered articles was the decision and planification techniques domain. Unlike the other subdomains, this one centers on the optimization of rail processes (i.e., optimization of flow transport, energy, fuel consumption) and various rail predictions (i.e., profit prediction, flow prediction, possible rail accidents). In fact, this domain can be subclassified as: rail transport optimization (74%), rail transport insights (16%) and energy optimization (10%) (see Figure 9).

**Figure 10:**
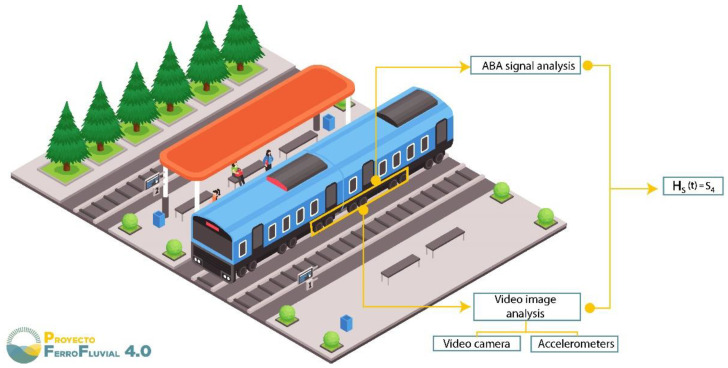
Physical architecture for obtaining train real-time data for a decision-support approach proposed in [108].

The mentioned subdomains are described below:

- *Rail transport optimization*: Studies classified in this subdomain proposed algorithms based on technologies of industry 4.0 in order to optimize railway traffic flow. The majority of the studies classified in this subdomain aimed to solve the following problems: prediction of train arrival times, minimization of train delay times, train rerouting process, train scheduling problem and the improvement of train dispatching systems. In fact, most of the studies focus on the prediction and minimization of train delays, which is an important research field. This is because trains are usually operated under a planned schedule timetable and sometimes the planned schedule cannot be accomplished due to external factors such as repair work, accidents or weather conditions. Therefore, the timetable has to be updated in order to decrease delay times in the train and prevent the delay propagation to other trains [80]. An example of the aforementioned articles is [85], where authors proposed an algorithm based on machine learning to predict an estimated arrival time of freight trains on the United States freight rail network. Specifically, authors used a Support Vector Regression Machine (SVM) [121] to predict the arrival times of freight trains using scalar features such as the train length, train tonnage, train horsepower per ton and train priority, among others. This solution presents an average improvement of 16% compared to deep learning solutions using the same dataset presented in their articles. It is worth mentioning that the algorithms oriented to solve the problem of minimizing train delay times can be divided in local-scale and large-scale algorithms. The majority of real-time rescheduling algorithms for train timetables are focused on solving local-scale railway systems due to the large amount of computational resources needed to estimate large-scale systems. Nevertheless, as computational resources continue to grow in their capacity and research improves the quality of the developed algorithms, various authors have developed innovative research to tackle this problem using one or multiple representations of Mixed Integer Linear Programming (MILP) models [122,123].
- *Rail transport insights*: This subdomain focuses on using railway collected information in conjunction with technologies such as big data, IoT or cloud computing to get insights that could improve railway processes. For example, in [108] a decision support approach is proposed using big data analysis to improve the rail maintenance process. They utilize a fuzzy-inference model to make rail maintenance decisions. They fed this model with real data taken from an intelligent rail condition monitoring and existing data on the track Amersfoort-Weert in the Dutch railway network. In Figure 10, we show the physical proposed architecture for this study.
- *Energy optimization*: This is a global issue that has been addressed by many researchers and governments in different fields. The identification of drivers and barriers to climate change mitigation in each study field is important since climate change cannot be tackled solely by optimizing the used resources in mechanical or technological components, and climate change affects transportation worldwide [124]. The train transportation field is no exception, and that is why the included papers in this subdomain focus on the train energy resources optimization. Specifically, the proposed approaches are related to energy optimization in the train operation, train timetabling and rolling circulation. In [116], authors proposed an optimization algorithm based on the Simulated Annealing (SA) algorithm to minimize the energy resources used by the train during their operation in the railroad. They used the SA algorithm to minimize train traction energy but constrained to an existing timetable for that train allowing them to optimize energy resources without altering the existing planned schedule.

From the identified domains in the SLR, the main issues in the railway transport industry are related to the development of monitoring tools. These must be able to get real-time data from physical elements such as trains, rail-track infrastructure and its components (sleepers, ballasts, railway tunnels and bridges). The development of these tools allows the early detection of damaged components improving the safety of rail tracks, decreasing maintenance costs and give proper advice to train drivers with the analyzed real-time data.

Another detected main issue is the minimization of train delays, which has been highly studied by researchers from multiple point of views such as traffic control, schedule adjustments and travel information systems [96]. This is a crucial problem that must be addressed because rail track systems are complex and there is a growing demand for freight and passenger train transportation.

Lastly, communication and security of the rail infrastructure is a growing study field among researchers. The scientific community has managed to use industry 4.0 technologies to develop protocols that can be applied to rail processes and infrastructure. These protocols are oriented to the train communication in hard-to-reach areas or are aimed at preserving the integrity of physical and digital spaces that are only accessible to authorized personnel.

It is important to highlight that many of these solutions can be applied to passenger and freight transportation problems. For example, there are proposed solutions oriented to tackling specific problems in each of these transportation modes. For example, in the freight transportation there are rail track systems exclusive only for this mode. Therefore, merging problems can be present when multiple freight trains are in congested areas or monitoring systems must be developed specifically for these trains. Solutions such as a train scheduling algorithm for only freight trains [26], monitoring systems for freight trains [60] or a DAS for merging freight trains in complex environments [53] are some examples. Additionally, there are solutions for only the passenger transportation mode such as a travel connectivity for train passengers [78], or for both modes such as a scheduling algorithm for freight and passenger trains [93] that is useful when rail tracks are shared.

### 3.2. Answer to the Second Research Question

Technological strategies in this research refer to the combination of technologies and methods that appeared in the reviewed articles for this SLR for each domain. It is important to clarify that this is a general perspective of what could be seen in the reviewed articles. In terms of the technology strategies that are currently being used to tackle the main issues described in Section 3.1, we identified the following:

#### 3.2.1. Monitoring Domain

Many of the monitoring domain solutions that were selected in this SLR use IoT technologies to capture real-time data. Then, the captured data is sent to processing algorithms (which in some cases are stored in the cloud) in order to clean it. Later, we identified that the core of the proposed solution implemented by the authors utilized algorithms based on AI, SDSS, CV and modelling and simulation. Finally, alerts are sent to the personnel to attend the incoming requirement if needed (i.e., check a rail component state).

Some of the identified algorithms for these industry 4.0 technologies are the following: Breadth First Search (BFS) algorithm or Genetic Algorithms (GA) for DAS systems [53,55], Principal Component Analysis (PCA) algorithm for monitoring rail breakage [18], Artificial Bee Colony (ABC) algorithm for a train traction control systems [57], Dynamic Differential Evolution (RHMDE) algorithm for tracking the rail state [10], fuzzy systems or deep-learning models for rail maintenance [28,44].

#### 3.2.2. Communication and Security Domain

This is an emerging domain in the railway industry from which we obtained a few articles. From the analyzed studies, we could not identify a specific strategy for the solutions, but most of them used IoT devices in their architecture. Many of these solutions use Machine Learning (ML) or Deep Learning (DL) models in conjunction with sensors, cameras, optical fibers or even GPS to improve security and communication protocols in train travels [78], restrict the access to train station areas [75], detect an overcrowding level risk in train stations [65] or establish cybersecurity measures for the implemented digital systems [77].

#### 3.2.3. Decision and Planification Techniques Domain

Lastly, the solutions grouped in this domain present two principal strategies. The first one is aimed at obtaining insights from railway data that were previously stored in databases while the second one focuses on the use of real-time data in the proposed solution. It is crucial to mention that the identified papers include different data preprocessing steps for each solution. In addition, in the case of real-time data, these are obtained by using IoT devices installed in trains, railroads or train stations such as the strategy introduced in the monitoring domain. Later, these data can be used in the proposed algorithms where they mainly focus on the optimization of railway processes via data analytics or the optimization of the railway traffic flow by using AI or modelling and simulation solutions.

Specifically, the identified industry 4.0 technologies used in this domain are AI, IoT, modelling and simulation, Big Data, SDSS, VR and Cloud Computing. Within these technologies we found the following algorithms for rail transport optimization: GA [104], Gaussian Kernel Ant Colony Optimization (GKACO) algorithm [103], MILPs [93,97,102], PCA algorithm [100], ML [86,96] and DL [87,89] techniques. For obtaining railway insights, we identified the following tools based on big data and cloud computing: Apache Hadoop, Tashi Cloud Middleware [111], Apache Spark and Google Cloud Infrastructure [79].

### 3.3. Answer to the Third Research Question

In the studied solutions in this SLR, we could identify 9 technologies related to industry 4.0 and they can be classified as follows: (i) Artificial Intelligence (AI), (ii) Cloud Computing, (iii) Big Data, (iv) Internet of Things (IoT), (v) Cybersecurity, (vi) Simulation, (vii) Smart decision support systems (SDSS), (viii) Computer Vision (CV) and (ix) Virtual Reality (VR). The identified studies belonging to these technologies are compiled in Table 5.

It is important to highlight that the selected papers can be classified in multiple types of industry 4.0 technologies. For example, we identified that solutions oriented to the railway industry are usually validated with computational simulation or real data. In order to capture real data, they use machine sensors installed in the train or the rail track and, for example, big data procedures to process it.

**Figure 11:**
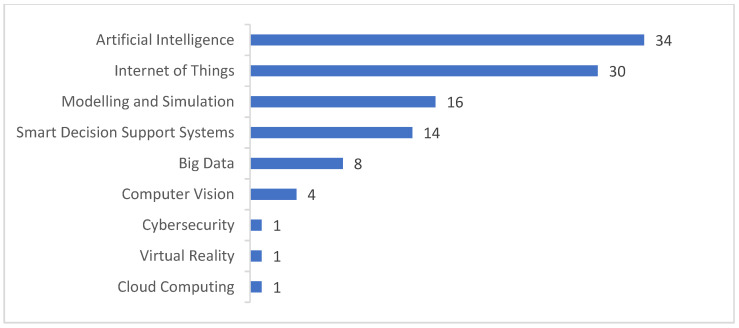
Number of articles per industry 4.0 technology.

As shown in Figure 11, artificial intelligence is the most cited technology for train transport, followed by IoT solutions. In the train transport industry, AI applications include methods based on machine learning and deep learning being used for a diversified variety of problems. Principally, AI can be used for rail-track assignment [104], tackling the train-delay propagation problem [87], prediction of train flow [82], time-arrival prediction [86], rail-condition evaluation and management [17], fault detection in railway infrastructure [12], energy optimization [115], surveillance systems [76] and detection of strange objects in railroads [64].

Interestingly, there are some AI applications in this field that proposed strategies based on images when the problem is not directly an image problem. For example, authors in [87] proposed a method to tackle the delay propagation problem using train timetables as images. Specifically, the strategy they proposed is to represent the train events in the timetable as pixels of the image. Then, they use convolutional neural networks to extract patterns from this constructed image to identify relationships between train events and mitigate delay times.

The second most-cited technology in the train transport field is the internet of things. This technology is commonly used for designing monitoring systems for the railway infrastructure [59]. The IoT devices usually used in this field are sensors (e.g., Acoustic Emission Sensors, Matrix Based Tectile Surface Sensors, etc.), radars, embedded computers, wearables, arduinos, Global Positioning Systems (GPSs) and cameras. This technology allows the capture of real-time data of the train, railroads and other railway infrastructure assets in order to convert the data into information that helps the improvement of the railway processes. The IoT technology has been used in multiple train transportation problems such as freight-train parameters monitoring [60], railway-tunnel structure monitoring [29], weather monitoring in railroads [30], train localization [63] and train dispatching systems [90].

On the other hand, modelling and simulation solutions have an important role in train transportation technologies. Usually, solutions created for this field are tested with real data through the use of realistic software simulators that allow them to tune the proposed methods and get conclusions about the research. In [84], authors proposed a method where they used a software simulator for train transport called Rail Traffic Controller (RTC) [133]. This simulator allowed them to study the relationship between trains that exceed the length of passing sidings and the train delays in single-track rail corridors.

Smart decision support systems are used in the train transport processes as a way to help operators’ and managers’ tasks via processed information collected from the railway system. DAS’s are a good example of support systems in the railway industry. They are used to provide optimized and detailed advice to train drivers. In [53], authors proposed a DAS to provide time/speed advice to freight train drivers in order to help them merge easily in mixed traffic corridors. They can be oriented to give proper advice in other topics such as train-trajectory optimization [55] or energy optimization [116].

The technologies that are not cited as often are Big Data, Computer Vision (CV), Cybersecurity and Cloud Computing. Big Data can be used in the railway transportation systems as a way of using Big Data parallel architectures to process the large amount of data collected from dynamic large-scale railway networks [79]. It can be mostly used for train-delay prediction [79] and condition-based maintenance systems [108,109]. Although CV is not cited often, it can be applied in the other industry 4.0 technology branches such as AI solutions. In [48], they developed a deep neural network for object detection using image processing and CV techniques that are also applied in railway defect detection, condition monitoring and diagnostics [28].

Finally, we have the least-cited technologies in the selected studies, which are Cybersecurity and Cloud Computing solutions. In this field, we found that cybersecurity is becoming an important technology to apply in the railway infrastructure due to the incremental use of IoT applications where a security access control scheme is of vital importance to preserve access only to authorized personnel [77]. Meanwhile, Cloud Computing solutions are also an emerging technology in the railway industry because they help solve the problems related to the storage of massive amounts of data coming from multiple sources and making them available for future analytics such as in [110], where a data-fusion system for railway infrastructure using cloud computing is proposed.

## 4. Limitations of This Study

The presented study was performed by extracting papers from two scientific digital library databases to synthesize the growing body of literature. However, this is one of the limitations of this study. It is highly possible that there are papers from other scientific digital library sources that can complement our findings and give a better overview of the actual research trends in this field. This can also be related to the selected keywords for the search algorithm that may have some bias for particular search interests. Nevertheless, other search algorithms based on the same or different keywords could be created and have the possibility of recovering works that were not considered in this review.

Another limitation of this study, as we mentioned in the results sections, is that many of the reviewed articles can be classified into multiple industry 4.0 technology groups. This is because the solutions are not limited to the use of only one type of technology; they can be combined to get a better and optimized approach to solve a problem. Therefore, authors classified the reviewed articles into the identified technology groups shown in Table 5 by using a personal perspective of the principal technology used in these papers. Nevertheless, other researchers can classify the same articles into other groups and results could change. Lastly, the protocol utilized in the methodology did not enforce a rigorous detailed analysis of the validity of the works, which could miss internal flaws in the study design and results from the articles reviewed.

## 5. Conclusions and Future Works

In this paper, we present a systematic literature review on the studies related to industry 4.0 technologies applied in the railway industry. With this, we aimed to recognize the most important problems in the railway industry and how these have been solved with industry 4.0 technologies. We started our SLR by establishing three research questions: what are the main issues in the railway transport industry, what are the technology strategies that are currently being used to solve these issues and what are the technologies from industry 4.0 used in the railway transport industry that are used to solve the aforementioned issues? To answer these questions, we reviewed the past 5 years’ studies oriented to solve rail transportation problems using industry 4.0 technologies by proposing multiple query algorithms that were used in two digital libraries named Science Direct and Web of Science from which we extracted the primary studies. Then, we filtered through them using inclusion and exclusion criteria to select the relevant studies to answer our research questions.

After reviewing the selected 109 primary studies, we were able to identify three main application domains: monitoring domain, decision and planification techniques domain, and communication and security domain. In addition, we presented multiple subdomains in which we tried to categorize the extracted primary studies based on a set of existing subproblems in the railway industry. Therefore, the monitoring domain has multiple subdomains that are rail monitoring, Driver Advisory Systems (DAS’s) and train monitoring; the decision and planification techniques domain has studies that are mostly oriented to rail transport optimization, rail transport insights and energy optimization; and lastly, the communication and security domain focuses its works on railway safety, security systems and travel connectivity problems.

Additionally, we identified nine industry 4.0 technologies that make contributions to tackle the existing problems in the railway industry: Artificial Intelligence (AI), Internet of Things (IoT), Cloud Computing, Big Data, Cybersecurity, Modelling and Simulation, Smart Decision Support Systems (SDSS), Computer Vision and Virtual Reality (VR). The identified technologies are not exclusive in their usage to propose a solution. In fact, multiple works show how some of these are related (i.e., AI and Computer Vision) and the combination can lead to better results or innovative solutions. This is not something that happens only in the rail industry for industry 4.0 technologies. The same effect can be seen in other fields such as the Smart Manufacturing Systems (SMS) in which the integration of these 4.0 technologies can be applied to the creation of semi-autonomous industrial systems [134,135].

Finally, additional studies should be performed by including other digital library sources in order to identify other approaches in which industry 4.0 technologies are involved in the rail transportation field. This can contribute to the number of identified articles and variety of approaches by subdomain in which fewer articles were classified (i.e., travel connectivity or energy optimization subdomains). In addition, other SLRs can be performed in this field to identify approaches comprising the environmental envelope and construction of railway infrastructure systems. Topics such as the development of more sustainable systems or the mitigation of environmental impacts have been addressed in some of the reviewed articles but require further exploration.

## Figures and Tables

**Table 1:** Information sources used for the search phase.

Data Source	Type	URL
Science Direct	Digital Library	[5]
Web of Science	Digital Library	[6]

**Table 2:** Keywords used for the search queries.

Group	Keywords
Group 1	Train transport, freight trains, railway system, passenger train.
Group 2	Rail monitoring, technology, driver advisory system, sensors, unmanned driving, train delay.
Group 3	Artificial intelligence, machine learning, deep learning, big data, internet of things, industry 4.0.

**Table 3:** Search query algorithms.

Digital Library	Group	Algorithm
Science Direct	Group 1 and group 2	TITLE-ABS-KEY ((“train transport” OR “freight trains” OR “railway system” OR “passenger train”) AND (“rail monitoring” OR “technology” OR “driver advisory system” OR “unmanned driving” OR “train delay”)) AND PUBYEAR > 2016
Web of Science	Group 1 and group 2	TITLE-KEY ((“train transport” OR “freight trains” OR “railway system” OR “passenger train”) AND (“rail monitoring” OR “technology” OR “sensors” OR “driver advisory system” OR “unmanned driving” OR “train delay”)) AND PUBYEAR > 2016
Science Direct	Group 1 and group 3	TITLE-ABS-KEY ((“train transport” OR “freight trains” OR “railway system” OR “passenger train”) AND (“machine learning” OR “deep learning” OR “big data” OR “internet of things”)) AND PUBYEAR > 2016
Web of Science	Group 1 and group 3	TITLE-KEY ((“train transport” OR “freight trains” OR “railway system” OR “passenger train”) AND (“artificial intelligence” OR “machine learning” OR “deep learning” OR “big data” OR “internet of things” OR “industry 4.0”)) AND PUBYEAR > 2016

**Table 4:** Clustering of the selected studies by subdomain.

Domain	Subdomain	Studies
Monitoring	Rail monitoring	[8,9,10,11,12,13,14,15,16,17,18,19,20,21,22,23,24,25,26,27,28,29,30,31,32,33,34,35,36,37,38,39,40,41,42,43,44,45,46,47,48,49,50,51,52]
	Driver advisory systems	[53,54,55,56]
	Train monitoring	[57,58,59,60,61,62,63,64]
Communication and security	Railway safety	[65,66,67,68,69,70,71,72]
	Security systems	[73,74,75,76,77]
	Travel connectivity	[78]
Decision and planification techniques	Rail transport optimization	[79,80,81,82,83,84,85,86,87,88,89,90,91,92,93,94,95,96,97,98,99,100,101,102,103,104,105,106]
	Rail transport insights	[107,108,109,110,111,112]
	Energy optimization	[113,114,115,116]

**Table 5:** Identified industry 4.0 technologies in the systematic literature review (SLR).

Technology	Description	Studies
Artificial Intelligence (AI)	Artificial Intelligence can be defined as a technology capable of developing thought processes like learning, reasoning, and self-correction similar to humans to supplement and increase worker capabilities [125].	[12,15,17,23,38,40,42,44,45,47,49,50,52,53,56,57,58,64,76,80,81,82,83,85,86,87,89,91,95,96,101,104,105,106]
Cloud Computing	Cloud computing is a model for enabling ubiquitous, convenient, on-demand network access to a shared pool of configurable computing resources (e.g., networks, servers, storage, applications, and services) that can be rapidly provisioned and released with minimal management effort or service provider interaction [126].	[110]
Big Data	The term big data has been created to describe the methods and techniques that process and extract meaning from very large amounts of data [66].	[66,79,94,107,108,109,111,112]
Internet of Things (IoT)	IoT is a unique system attaining rapid recognition in the world of contemporary wireless telecommunication. IoT consists of billions of devices, people, objects and services seamlessly communicating and exchanging information about themselves and their environment [127].	[11,20,21,24,25,27,29,30,31,32,33,34,35,36,37,39,41,43,46,59,60,61,62,69,73,74,75,78,90,115]
Cybersecurity	Cybersecurity means the activities necessary to protect network and information systems, the users of such systems, and other persons affected by cyber threats [128].	[77]
Modelling and Simulation	Modelling and simulation can be defined as a discipline that allows the creation of models that can approximate an event or a system from the real world. In conjunction with simulations, the created models can be modified and analyzed to get conclusions, verify and validate the research [129].	[18,26,63,72,84,88,92,93,97,98,99,100,102,103,113,114]
Smart Decision Support Systems (SDSS)	Smart decision support systems use learning and problem-solving techniques to solve complex problems in real contexts. They improve operator performance by providing detailed process optimization instructions [130].	[8,9,10,13,14,16,22,54,55,65,67,68,71,116]
Computer Vision	Computer vision can be defined as a technology for describing the world as humans see it in one or more images, reconstructing properties such as shape, illumination and color distributions [131].	[28,48,51,70]
Virtual Reality (VR)	Virtual reality (VR) is a technology that incorporates computer-generated, interactive and highly vivid environments that enable the user to achieve a state of immersion through the ultimate experience of telepresence, and facilitate engagements in human encounters that are multi-sensorial, dynamic and resemble the user’s perception and understanding of the real world [132].	[19]
